# Iron homeostasis in older adults: balancing nutritional requirements and health risks

**DOI:** 10.1016/j.jnha.2024.100212

**Published:** 2024-03-14

**Authors:** Rola S. Zeidan, Matthew Martenson, Javier A. Tamargo, Christian McLaren, Armin Ezzati, Yi Lin, Jae Jeong Yang, Hyung-Suk Yoon, Taylor McElroy, James F. Collins, Christiaan Leeuwenburgh, Robert T. Mankowski, Stephen Anton

**Affiliations:** a Department of Physiology and Aging, College of Medicine, University of Florida, Gainesville, FL, USA; b Department of Health Outcomes and Biomedical Informatics, College of Medicine, University of Florida, Gainesville, Florida, USA; c Department of Clinical and Health Psychology, College of Health and Health Professions, University of Florida, Gainesville, Florida, USA; d Department of Food, Nutrition, Dietetics and Health, Kansas State University, Manhattan, KS, USA; e UF Health Cancer Center, Gainesville, FL, USA; f Department of Surgery, College of Medicine, University of Florida, Gainesville, FL, USA; g Department of Food Science & Human Nutrition, Institute of Food and Agricultural Sciences, University of Florida, Gainesville, FL, USA

**Keywords:** Iron, Nutrition, Homeostasis, Aging

## Abstract

Iron plays a crucial role in many physiological processes, including oxygen transport, bioenergetics, and immune function. Iron is assimilated from food and also recycled from senescent red blood cells. Iron exists in two dietary forms: heme (animal based) and non-heme (mostly plant based). The body uses iron for metabolic purposes, and stores the excess mainly in splenic and hepatic macrophages. Physiologically, iron excretion in humans is inefficient and not highly regulated, so regulation of intestinal absorption maintains iron homeostasis. Iron losses occur at a steady rate via turnover of the intestinal epithelium, blood loss, and exfoliation of dead skin cells, but overall iron homeostasis is tightly controlled at cellular and systemic levels. Aging can have a profound impact on iron homeostasis and induce a dyshomeostasis where iron deficiency or overload (sometimes both simultaneously) can occur, potentially leading to several disorders and pathologies. To maintain physiologically balanced iron levels, reduce risk of disease, and promote healthy aging, it is advisable for older adults to follow recommended daily intake guidelines and periodically assess iron levels. Clinicians can evaluate body iron status using different techniques but selecting an assessment method primarily depends on the condition being examined. This review provides a comprehensive overview of the forms, sources, and metabolism of dietary iron, associated disorders of iron dyshomeostasis, assessment of iron levels in older adults, and nutritional guidelines and strategies to maintain iron balance in older adults.

## 1 Introduction

Iron is an essential mineral that plays a crucial role in many physiological processes. Specifically, iron is indispensable for hemoglobin and myoglobin production, the production and function of several cellular enzymes and complexes (such as mitochondrial electron transport chain complexes), the regulation of immune function, as well as maintenance of healthy skin, hair, and nails [1]. Consequently, the human body needs to maintain steady levels of iron, which is achieved through a complex system of absorption, recycling, storage, and transport. Humans’ only source of iron is through dietary intake [2]. Iron absorption from food occurs mainly in the proximal small intestine and is tightly regulated [3]. The body also recycles iron from senescent red blood cells [4]. Excess iron is stored mainly in the liver, spleen, and bone marrow [3]. Interestingly, the human body has no active mechanism for excreting excess iron, so it relies on regulating absorption to maintain physiological iron balance [5].

Age can have a profound impact on systemic and cellular iron levels, eventually leading to a disruption of iron homeostasis [6]. Several age-related disorders can occur when iron levels increase or decrease beyond cellular threshold [7]. This strongly suggests that iron intake levels need to be carefully maintained in older adults to reduce the risk of organ dysfunction and development of pathologies. Therefore, it is prudent for older adults to follow recommended daily intake levels and consult with a healthcare provider before taking iron supplements.

In this review, we discuss, the physiological importance and mechanisms of maintaining iron homeostasis, as well as the effect of age on iron homeostasis and age-induced iron-related pathologies. We will also discuss the nutritional forms of dietary iron and recommendations for iron intake.

## 2 Methodological considerations

While not systematic in nature, a search strategy was still employed to identify relevant literature. Three electronic databases, including PubMed, Google Scholar, and Scopus were searched using appropriate keywords for each section. The search was not limited by publication date, but only articles published in English were included. Given the narrative nature of this review, articles were screened based on their relevance to the topic rather than strict inclusion and exclusion criteria. The search was content-led; however, inclusion criteria encompassed studies that provided valuable insights, theoretical frameworks, or empirical evidence related to the current review and specific to each manuscript section. Exclusion criteria included studies lacking relevance or those with inadequate methodological rigor according to the authors’ judgement. Other references were obtained through the literature cited.

## 3 Physiological iron homeostasis in older adults

### 3.1 Physiological uses of iron

Iron is a fundamental nutritional element since it is indispensable for several physiological functions. For example, iron is a critical component of several heme proteins, including hemoglobin, myoglobin, cytochromes, and iron-sulfur clusters [8]. Hemoglobin is composed of four protein subunits, each containing a heme group that binds to an iron ion [9]. Iron is vital to produce heme (for hemoglobin), which is synthesized in developing red blood cells in the bone marrow [10]. Hemoglobin accounts for around two-thirds of body iron and is responsible for transporting oxygen from the lungs to peripheral tissues and organs [11]. Myoglobin is another heme protein that binds to oxygen and contains about 15% of total body iron. Myoglobin is found in muscle tissue and helps store oxygen for use during exercise or periods of low oxygen availability [11]. Additionally, iron is a critical component of cytochromes, which are involved in the electron transport chain, contain heme, and play a role in energy production (ATP, adenosine triphosphate) within cells [8].

Furthermore, iron plays a crucial role in DNA synthesis, DNA repair, and cell cycle control by being an important component or a cofactor of enzymes involved in these processes [12]. Iron deficiency can impair DNA synthesis and repair, and cause cell cycle arrest. This can have serious consequences, particularly in rapidly dividing cell populations (e.g., blood, skin, and immune cells), leading to health problems, such as skin rashes and impaired immune function [[12], [13], [14]].

Additionally, iron is essential for proper immune function where it is required for the proliferation and differentiation of immune cells, such as lymphocytes, macrophages, and neutrophils [15]. Iron deficiency can thus compromise immune function [14]. Moreover, iron is a key player in ferroptosis, a type of cell death characterized by increased iron-induced oxidative stress, which plays a critical role in immune surveillance (the process by which immune cells recognize foreign pathogens) and tumor suppression [16,17]. Impaired ferroptosis, however, can be aggravated by insufficient nutritional iron intake and can contribute to the development of multiple pathologies including neurodegeneration, acute organ damage, ischemic reperfusion injury, and cancer [18,19].

Collectively, it is evident that iron is indispensable for various physiological functions. There are adverse consequences of elevated or low cellular (and systemic) iron. Older adults, in particular, are susceptible to neurodegenerative diseases, cardiometabolic diseases, and cancer, and risk for all of these conditions is influenced by iron imbalance. It is thus crucial that physiological iron balance is maintained in older adults.

### 3.2 Iron homeostasis

**Fig. 1:**
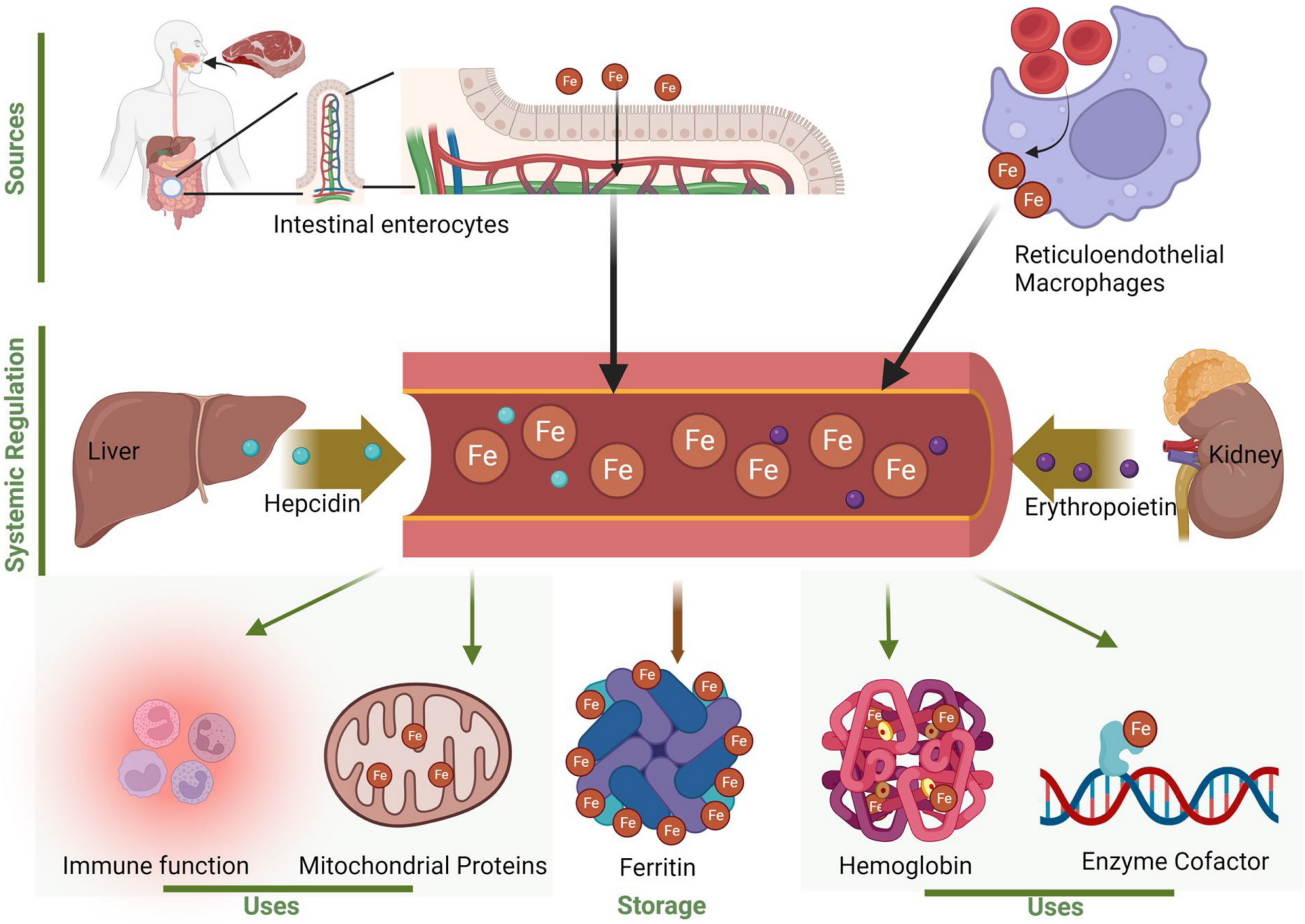
This figure provides a comprehensive overview of the sources of biological iron, the processes that systemically balance iron levels, and the cellular uses of iron.

Iron homeostasis is maintained through a complex system of regulation, which ensures that systemic iron levels remain within a safe range [8]. The regulation of iron homeostasis involves the absorption, transport, storage, recycling, and utilization of iron [5] (Fig. 1). Iron excretion is very limited and occurs primarily through the gastrointestinal tract, which occurs at a steady rate irrespective of systemic iron levels [8], although new research suggests that excess iron might be excreted through bile [20]. Additionally, a small amount of iron might also be excreted through sweat, urine, and menstrual blood [21]. Therefore, the major regulation of iron levels occurs at the absorption level, which takes place primarily in the duodenum and upper jejunum of the small intestine by intestinal enterocytes [5,22].

*Absorption*: Iron transport varies according to the type of iron. Non-heme ferric (Fe^3+^) iron is absorbed by intestinal enterocytes after reduction by the ferrireductase duodenal cytochrome B (DCYTB) to the ferrous state (Fe^2+^), via import by divalent metal-ion transporter DMT1, a ferrous iron/proton cotransporter [23]. Enterocyte basolateral membrane ferroportin (FPN, the only known cellular iron exporter) exports iron into the blood, where it is transported by transferrin, a glycoprotein that binds and transports iron through blood plasma [8]. Transferrin binds to and transports iron through the bloodstream to most cells [8]. Diferric (or *holo*-) transferrin binds to transferrin receptor 1 (TFR1) and is endocytosed into the cell, followed by iron export from endosomes into the cytosol [8]. Details of how the other main dietary form of iron, heme iron, is absorbed and assimilated by intestinal enterocytes remain unclarified, but probably involve receptor-mediated endocytosis [23]. Different forms of dietary iron thus enter the cytoplasm by different mechanisms, and can subsequently be utilized within the cell, stored in ferritin or exported by FPN [24]. For a comprehensive elucidation of the intricacies pertaining to cellular and systemic iron homeostasis, we direct readers to consult our comprehensive review on this subject matter [8,22].

*Utilization and Storage*: Systemically, iron is mainly stored in the liver (hepatocytes, Kupffer cells), spleen (macrophages), intestine (enterocytes for short-term storage) and bone marrow, and is released when needed [23]. At the cellular level, in the cytoplasm, iron is utilized by the cell or stored in ferritin, which is a complex protein that can store several thousand iron atoms mainly in enterocytes, macrophages, and hepatocytes [8]. The cytoplasmic labile iron pool (LIP) intermediates between imported and stored and utilized iron in the cell [8,25]. Iron storage maintains LIP levels low, and therefore limits the potential for free-iron mediated oxidative stress [23]. Mitoferrin (MTFN1) imports cytosolic iron into mitochondria, where it is utilized for heme production, iron-sulfur cluster biosynthesis, or stored in mitoferritin (MtFt) [8]. Iron is also transported to the nucleus where it functions as a component or a cofactor for several DNA repair, synthesis, and transcription enzymes and factors as previously mentioned [12,26].

*Homeostasis and Regulation*: In healthy, young individuals, iron levels are intricately maintained at both systemic and cellular levels, with cross-talk between the two [8]. With aging, however, these processes become dysregulated (as described below). The liver plays a key role in regulating iron levels systemically [23]. Specifically, hepatocytes secrete the hormone hepcidin to reduce intestinal iron absorption and iron release from stores in reticuloendothelial macrophages (which recycle iron from senescent red blood cells they digest) when iron levels are high [27]. In contrast, hepatocytes prompt the release of iron from ferritin when iron levels are low [23].

### 3.3 Age-induced iron dyshomeostasis

Organismal aging presents multiple challenges to the regulation of iron homeostasis. Specifically, there is evidence that the aging process can be associated with both iron overload and iron deficiency.

*Iron Overload*: Iron accumulation in parenchymal tissues increases with age, potentially due to excessive iron intake, genetic factors, or certain pathological conditions, including thalasemia, hemosiderosis, aplastic anemia, and ineffective erythropoiesis [28]. Primarily, hepcidin levels appear to be upregulated with inflammaging (i.e., age-related chronic inflammation), leading to enhanced cellular iron retention [23]. Moreover, increased intestinal permeability, colloquially referred to as "leaky gut," is observed in the older adult population and may potentially contribute to a systemic elevation in the pathological non-transferrin-bound iron [23]. This could be aggravated by dysbiosis of the gut microbiota in older adults. Oxidative stress and cellular damage can result when age-related non-heme iron accumulation exceeds iron storage capacity. Excessive iron (Fe) at the molecular level can precipitate the Fenton reaction, increasing reactive oxygen species (ROS), and ultimately leading to damage in biological macromolecules [29]. One overarching feature across all tissues is the effect of iron accumulation in the mitochondria. Defects in the biosynthesis of heme and iron-sulfur clusters may lead to age-dependent non-heme iron accumulation in mitochondria, which can increase mitochondrial dysfunction and cellular damage [30] leading to clinical consequences in the vasculature [31], skeletal muscle [28,32], and neuronal tissue [33], as well as in several other organ systems (as described in Table 1).

**Table 1:** Iron dysregulation-related organ dysfunction. This table summarizes the mechanisms, associated pathologies, and symptoms of iron-related disorders in various organ systems. Iron overload and iron deficiency have distinct effects on organ systems, leading to oxidative stress, inflammation, and fibrosis in some cases, and reduced enzyme activity or hormone production in others.

Organ system	Mechanism	Associated pathologies	Associated symptoms
Liver	Iron overload-induced hepatic fibrosis; iron deficiency reduces activity of cytochrome P450 enzymes involved in drug metabolism	Liver cirrhosis, hepatic-hypoxia	Fatigue, abdominal pain, joint pain, weakness, liver damage, diabetes mellitus
Kidneys	Excess iron accumulation causes iron-mediated oxidative stress and subsequent renal fibrosis; leading to impaired renal cell function.	Chronic kidney disease	Kidney damage, decreased kidney function
Brain	Excessive iron accumulation has been linked to increased risk of neurodegenerative diseases such as Parkinson's and Alzheimer's diseases, iron-induced oxidative stress and lipid peroxidation, potentially causing neuronal damage and impairment of their functions	Parkinson's disease, Alzheimer's disease	Memory loss, tremors, impaired motor function
Heart	Iron is involved in oxygen transport and utilization; iron overload causes oxidative stress, inflammation, and fibrosis	Atherosclerosis	Fatigue, shortness of breath, chest pain, heart damage
Pancreas	Iron-induced oxidative stress and the formation of advanced glycation end products (AGEs) impair insulin signaling and contribute to insulin resistance; iron overload causes beta cell dysfunction and impaired insulin secretion	Type 2 Diabetes mellitus	Increased thirst and hunger, fatigue, weight loss, abdominal pain
Joints	Iron is a component of several enzymes and proteins involved in cartilage and bone formation, maintenance, and repair; iron deficiency leads to reduced collagen synthesis and impaired bone formation; iron overload leads to joint damage and inflammation	Osteoarthritis, joint pain	Joint pain, stiffness, reduced mobility
Endocrine glands	Iron deficiency leads to decreased hormone production and secretion	Hypothyroidism, sexual dysfunction, infertility	Fatigue, weakness, weight gain, decreased libido

*Iron Deficiency*: Iron deficiency is also prevalent among older populations, with anemia being the most common manifestation of severe iron deficiency [23]. In the United States, approximately 10% of females and 11% of males aged 65 years and older are anemic; however, the prevalence doubles in adults aged 85 years and above [34,35]. Data from the National Health and Nutrition Examination Survey (NHANES) (1988–1994) suggested that roughly 20% of all anemia in older adults was related to iron deficiency [36]. Anemia can have significant repercussions for older adults, especially in terms of their physical and cognitive functioning [31,35,37,38]. Anemia potentially impacts the vasculature through an unclear mechanism that dysregulates vascular smooth muscle cell cytoskeletal integrity [39]. Although less clear than the well-established iron deficiency anemia, non-anemic iron deficiency also has clinical relevance in conditions such as heart failure and chronic obstructive pulmonary disease [40,41]. Similar to iron deficiency anemia, non-anemic iron deficiency in older adults is also associated with reduced physical performance and increased mortality [42]. Non-anemic iron deficiency in older adults is possibly caused by inadequate dietary intake or low iron bioavailability, impaired iron absorption, or excessive iron losses [42].

Given that low-grade inflammation is common in older adults [43], elevations in inflammatory cytokines, such as IL-6, can lead to increased levels of hepcidin. Hepcidin inhibits iron release from cells that absorb (enterocytes) and store (Reticuloendothelial RE macrophages) iron [44]. Thus, inflammation-mediated hepcidin increases can potentially contribute to both anemic [often referred to as the anemia of inflammation (AI) or the anemia of chronic disease (ACD)] and non-anemic iron deficiency. Consequently, aging-induced dysregulation of iron clearly plays an important role in pathologies associated with aging [45].

## 4 Age-induced, iron-related pathologies

Iron imbalance, including both overload and deficiency, causes various pathologies that affect overall well-being. Iron overload results from excessive iron accumulation over time. This accumulation can be caused by underlying medical conditions that require chronic blood transfusion, in chronic liver disease, mutations in genes encoding iron-regulatory proteins, or excessive iron intake [28]. Iron overload leads to a wide range of symptoms and complications depending on the duration and extent of iron accumulation. Conversely, iron deficiency and subsequent anemia are commonly observed in older adults, resulting from inadequate intake, poor absorption, or increased iron losses [39]. Both excessive iron levels and insufficient iron levels can disrupt normal physiological processes, resulting in dysfunction across multiple organ systems (Table 1) and increasing susceptibility to age-related chronic diseases [1]. This is of special interest to the aging population, particularly concerning common age-related neurological and cardiometabolic diseases, which we will discuss in this section.

### 4.1 Neurodegenerative diseases

Recent work has suggested that the dysregulation of metal-ion homeostasis, such as iron, zinc, copper, and manganese, is associated with the pathogenesis of neurodegenerative disorders such as Alzheimer’s disease (AD) and Parkinson’s disease (PD) [46]. Magnetic resonance imaging (MRI) studies consistently show elevated brain ferritin-bound iron levels in individuals with AD and PD [47,48], triggering disrupted cellular iron distribution and accumulation, and increasing the risk of developing these diseases [49]. The accumulation of iron can interfere with the synthesis of neurotransmitters, the production of myelin, and the activity of mitochondria [50]. Furthermore, this is especially important to older adults, as current research evidence suggests that age-related iron sequestration in neurons and glial cells can potentially lead to an increased risk for the development of AD and PD [[47], [48], [49]].

Elevated brain iron levels have been shown to increase amyloid precursor protein (APP) translation, which in turn promotes the aggregation of amyloid-beta (Aβ) and hyperphosphorylation of tau, which are pivotal factors in AD pathology [51,52]. Iron intake has also been associated with AD pathology in epidemiological studies. For example, a study based on the NHANES data found associations between excessive iron intake and increased risk of dementia in males, while suboptimal iron intake is associated with a higher risk of dementia in females [53]. Other studies have linked higher serum iron levels to reduced cognitive functioning [53]. This suggests that adequate iron consumption, such as limiting consumption to the recommended dietary allowance, may reduce the relative risk of AD pathology.

In the context of PD, both iron deposition and impaired cellular iron storage in the substantia nigra has been associated with motor dysfunction and altered iron levels that impair the function of dopaminergic neurons [48]. Studies have shown that increased dietary iron intake as well as increased iron supplementation. Can increase the risk of PD [54], especially with simultaneous intake of manganese which can double the risk [55]. Nonetheless, other studies have interestingly shown opposing results where decreased serum iron levels were associated with an increased risk in PD pathology, since certain cases of anemia are associated with an increased risk of PD [56,57]. These observations may imply a U-shaped response, wherein both low and high serum iron levels could contribute to an elevated risk of PD. Notably, studies investigating the effect of iron chelation therapies on neurodegenerative diseases have been largely inconclusive [58,59]. Considering these varied findings, personalized recommendations for iron intake are advised. Also, generally, monitoring manganese levels is recommended to reduce the risk of PD, in cases of iron supplementation.

### 4.2 Cardiometabolic diseases

Recently, iron has been implicated in the pathogenesis of cardiometabolic conditions commonly observed in older adults, including Type 2 Diabetes mellitus (T2DM) and atherosclerosis [60]. These findings are corroborated by the extensive research on iron overload, which has revealed its detrimental effects on vital organs such as the heart, pancreas, and liver [[61], [62], [63]]. Dysregulation of iron homeostasis disrupts glucose metabolism and can lead to insulin resistance, lipolysis, and inflammation, thereby contributing to the development and progression of related conditions [64,65]. However, the underlying mechanisms are still debated. In addition, age-related changes in inflammatory responses and organ function further potentiate the impact of iron dysregulation on the cardiometabolic health of older individuals [66].

The accumulation of iron can promote endothelial dysfunction, lipid accumulation, and insulin resistance via iron-induced redox reactions [67,68]. In the context of diabetes and atherosclerosis, iron exacerbates the burden of these diseases during aging [69]. Epidemiological evidence suggests a causal link between elevated iron stores and serum ferritin levels and the risk of the development of diabetes and atherosclerosis.

Iron sequestration within hepatocytes can dysregulate several cellular processes and impair iron storage and transport [70]. Consequently, this dysregulation triggers increased production of hepcidin, resulting in iron retention within pancreatic and macrophage cells [70]. Notably, even in the absence of significant iron overload, elevated body iron stores have been associated with glucose intolerance and the development of T2DM [64,71]. Within pancreatic islet β-cells, the accumulation of iron instigates inflammation, heightened production of ROS, and subsequent β-cell dysfunction, contributing to insulin resistance and the pathogenesis of T2DM [72]. In addition to elevated body iron stores, higher heme iron intake via the diet has been linked to a greater risk of T2DM [73].

In the case of atherosclerosis, iron overload in vascular cells leads to the production of reactive oxygen species, induction of apoptosis, and potential cardiovascular dysfunction, emphasizing the relevance of iron dysregulation in older adults [74]. Noteworthy, both iron deficiency and iron overload pose substantial cardiovascular risks. Systemic iron deficiency often coexists with cardiovascular disease, thereby increasing the susceptibility to coronary artery disease, myocardial infarction, and mortality in individuals with established coronary artery disease [74].

### 4.3 Cancers

Several studies have investigated the association between dietary iron intake and the risk of different types of cancer. The findings have been inconsistent, with some studies suggesting a positive association, while others reporting no significant relationship or even a protective effect [75,76]. Notably, many studies often rely on self-reported dietary intake data, which may be prone to recall bias and other limitations.

Iron accumulation has been implicated in the development and progression of several types of cancers. While one meta-analysis reported that an increase in heme iron intake was associated with an increased risk of developing some types of cancer (namely colorectal, colon, breast, and lung cancers but not for other cancers) [77], other meta-analyses have shown similar results (a significant positive association of diets high in heme iron with the risk of breast [78], colorectal [79], and esophageal [80] cancers but a null association with lung cancer [81]). On the other hand, other studies have shown that high intakes of total iron or supplemental iron rather significantly decreased the risk of certain cancer types (namely colorectal [79], esophageal [80], and gastric cancers [82]). Conflicting findings have been proposed in relation to lung cancer, where one study proposed that a diet rich in heme iron might decrease the likelihood of developing lung cancer, and another study indicated that the intake of heme iron was linked to an elevated risk of lung cancer, with a contrasting association observed for non-heme iron [83,84]. The latter study also showed a reduced risk of colorectal cancer related to diets high in non-heme iron among men [84]. Another epidemiological study reported a non-significant positive association between heme iron intake and pancreatic cancer [85]. However, this association became stronger and more significant when restricted to heme iron from red meat, especially among men. Possibly, iron accumulation, particularly in the mitochondria, has been implicated in the development of pancreatic cancer [86], although other research studies have revealed no association between hemochromatosis and pancreatic cancers [87]. Notably, the results from 16 years of follow-up on more than half a million middle-aged and older Americans, revealed that heme iron intake was linked to increases in all-cause and cancer-specific mortality [88].

There are a few potential mechanisms by which high iron intake could promote cancer development. First, iron accumulation increases oxidative stress through the Fenton reaction, leading to DNA damage and mutations contributing to the initiation and progression of cancer [89,90]. Additionally, iron homeostasis and inflammation are closely interconnected, and therefore iron imbalance can increase inflammation which can increase the risk of developing cancer [91]. Furthermore, iron is involved in various cellular processes that are dysregulated in cancer, such as cell proliferation, angiogenesis, tumor cell survival and reprogramming, and metastasis [91]. Excess iron may fuel these processes and promote tumor growth [90,92].

Taking all evidence into account, a positive association between heme iron intake and certain types of cancers could be indicated, but there is limited explanation on the association of dietary iron intake with carcinogenesis, tumor progression, as well as iron’s role in cancer development and prognosis among older populations. This warrants further research.

## 5 Assessment of Iron Levels in Older Adults

Considering the importance of maintaining homeostatic physiological iron levels, it is highly advisable to conduct regular screenings to assess iron status in older adults [93]. As previously mentioned, older adults are at risk of both iron deficiencies and iron overload [75,83,84,93,94]. Among older adults from the Framingham Heart Study, nearly 13% had elevated serum ferritin (SF) levels (>300 μg/L in men and >200 μg/L in women) suggestive of iron overload, and 3% showed evidence of iron deficiency (≥2 of 3: SF < 12 μg/L, TSAT < 15%, and Mean Corposule Volume - MCV < 80 fL) [93]. Nevertheless, there is a lack of consensus on guidelines for the diagnosis and treatment of iron deficiency, as differing guidelines have been developed and adopted by a wide variety of professional organizations [94,95]. Moreover, there is a need for updated screening guidelines for iron deficiency and iron-deficiency anemia, as existing guidelines are outdated [95]. Age-specific guidelines that correspond to older adults are desperately needed, as many biomarkers of iron status are affected by underlying health conditions that become increasingly common with older age [96].

**Fig. 2:**
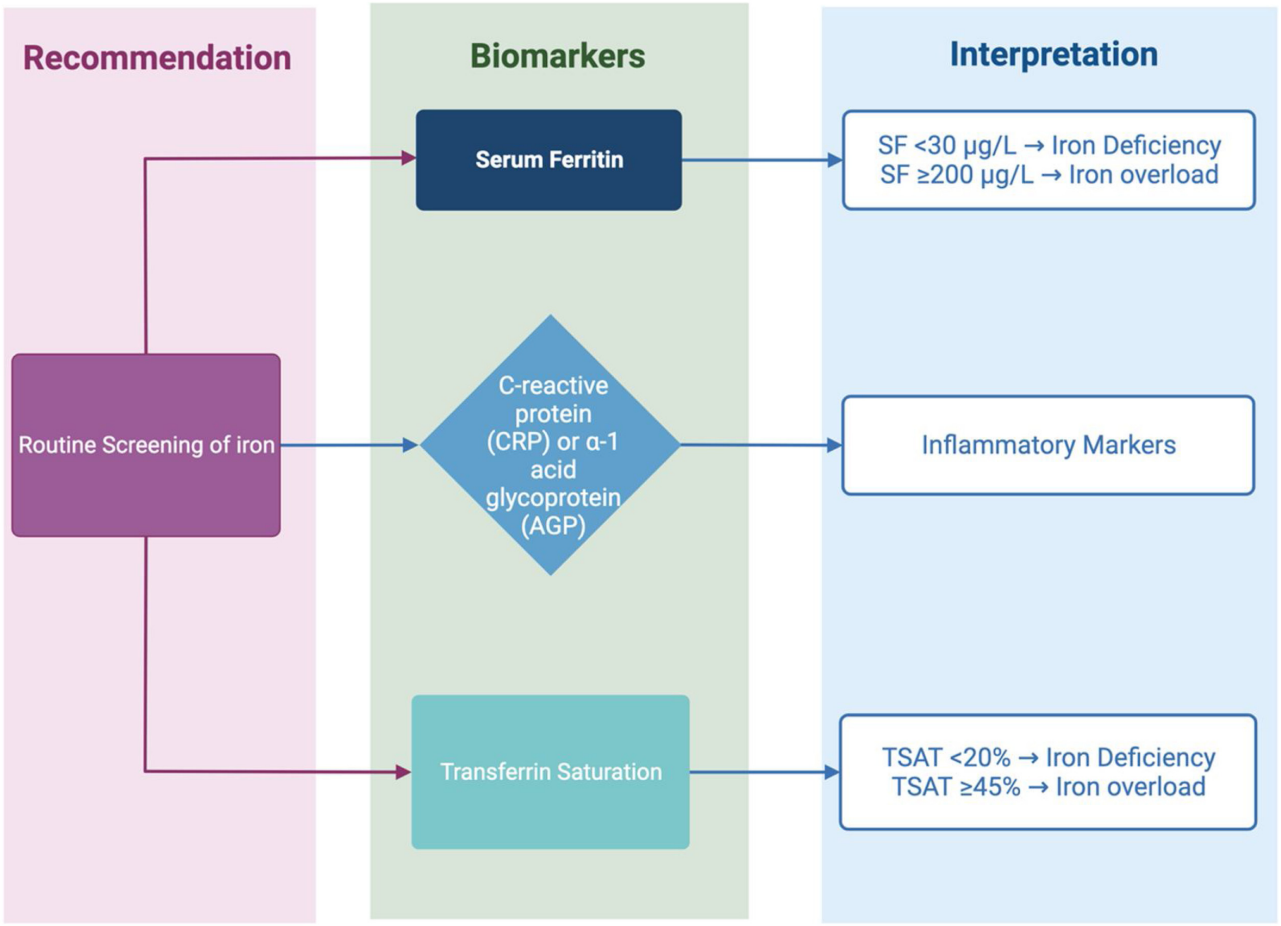
Routine screening of iron status is recommended for older adults. Serum ferritin (SF) and transferrin saturation (TSAT) are the most useful and easily obtained bioindicators of iron status. SF < 30 μg/L or TSAT < 20% are likely indicative of iron deficiency in older adults. SF ≥ 200 μg/L or TSAT ≥ 45% may be indicative of iron overload in older adults without inflammation or chronic disease that may account for elevated levels. Concurrent measurement of inflammatory markers C-reactive protein (CRP) or α-1 acid glycoprotein (AGP) is recommended.

Notably, assessment of iron status requires evaluation of several biochemical tests, as there is no single diagnostic test for altered iron status. The relationships between different biomarkers of iron status and different stages of iron deficiency or overload are shown in Table 2. The most widely used biomarkers are described below, along with guidance on the identification of iron deficiency and iron overload. A summary of the most widely used biomarkers for iron status assessment is represented in Fig. 2.

**Table 2:** Biomarkers of iron status in relation to stage of iron deficiency or iron overload.

Biomarker	ID stage 1: Iron depletion	ID stage 2: Erythropoiesis	ID stage 3: Anemia	Iron overload
Stainable bone marrow iron	↓↓	↓↓	↓↓	− or ↑
Serum ferritin	↓	↓	↓	↑
TSAT	–	↓	↓	↑
Erythrocyte Protoporphyrin	–	↑	↑	–
sTfR	–	↑	↑	–
Hemoglobin	–	–	↓	–

Symbols: high (↑), normal (−), low (↓), severely depleted or absent (↓↓).

Modified/adapted from Refs. [109,150].

Abbreviations: ID, iron deficiency; sTfR, soluble transferrin receptor; TSAT, transferrin saturation.

### 5.1 Serum ferritin (SF)

Serum ferritin concentrations correlate with whole-body iron stores, making it a viable marker of iron status in most cases (only in the absence of inflammation of other physiological stressors). It is the most common assessment indicator of iron status [94], as it is easily accessible, inexpensive, and is considered the most specific measure of iron deficiency.

#### 5.1.1 Iron deficiency

The only significant cause of low ferritin concentrations in serum is iron depletion, therefore a low serum ferritin test result has a high specificity for iron deficiency [97,98]. Also, reduced levels of serum ferritin precede morphologic changes in red blood cells (RBCs) or anemia; thus, it is a valuable tool in the early detection of iron-deficiency anemia. Furthermore, serum ferritin levels may increase as a result of inflammation, infection, or in certain chronic conditions, including hemochromatosis, chronic liver disease and hemolytic anemia, potentially masking iron deficiency. Specifically, both the Centers for Disease Control and Prevention (CDC) and the World Health Organization (WHO) recommend that serum ferritin levels <15 μg/L be considered an indicator of iron deficiency [99]. However, a cutoff of <30 μg/L has been more widely used since it provides higher sensitivity while maintaining specificity [100,101]. In cases of suspected infection or inflammation, the WHO recommends concurrent measurement of C-reactive protein (CRP) or α-1 acid glycoprotein (AGP), and a serum ferritin cutoff of <70 μg/L [100]. In anemic older adults, serum ferritin levels below 45−50 μg/L can be indicative of iron deficiency [101,102]. In patients with chronic inflammatory conditions, an international expert panel recently recommended the use of SF < 100 μg/L as a cutoff for iron deficiency (with or without anemia) [103]. If SF is between 100 and 300 μg/L, a transferrin saturation test is recommended to confirm iron deficiency.

#### 5.1.2 Iron overload

On the other hand, in the case of iron overload, the WHO recommends that serum ferritin levels ≥200 μg/L for apparently healthy men and non-menstruating women, or ≥500 μg/L in non-healthy adults be considered at risk of iron overload [100].

### 5.2 Transferrin saturation (TSAT)

TSAT is proposed as a test complementary to serum ferritin for assessment of iron status [94]. Transferrin saturation is the percentage of transferrin that is saturated with iron, and is calculated as the ratio of serum iron to total iron-binding capacity (TIBC) via the formula: TSAT = (Serum iron [μmol/L])/(TIBC [μmol/L])*100, and reported as a percentage. Transferrin saturation is considered a more sensitive bioindicator of iron deficiency than serum iron or TIBC. Serum iron levels can vary considerably as a result of taking iron-containing supplements, dietary intake, and normal diurnal variation. Total iron-binding capacity is unaffected by diurnal variation and is inversely proportional to body iron stores; it is elevated when iron is depleted and decreased in iron overload. It is generally recommended that a TSAT test is ordered along with serum ferritin in order to avoid burden, inconvenience, and cost to patients. Guidelines by the CDC and WHO suggest that TSAT of <15% or <16%, respectively, be considered indicative of iron deficiency [97,99]. In patients with chronic inflammatory disease, a TSAT < 20% is recommended [103,104]. Others have recommended TSAT < 25% as a cutoff for iron deficiency in anemic older adults [105]. On the other hand, TSAT levels of ≥45% in males and ≥40% in females are suggestive of iron overload, while levels below those thresholds can rule out iron overload even if ferritin is elevated [106]. An elevated TSAT may precede increased ferritin in iron overload; thus, an elevated TSAT with normal ferritin suggests a risk of iron overload.

### 5.3 Soluble transferrin receptor (sTfR)

Transferrin receptor (TfR) is expressed in virtually all cell membranes as it mediates cellular iron uptake [107]. Measurement of sTfR, a circulating form of the transferrin receptor protein after cleavage from cell membranes, has been proposed as a more sensitive indicator of iron deficiency, particularly in inflammatory or chronic disease states. Expression of TfR is increased in accordance with iron demands, such as in iron deficiency and erythropoiesis. As such, sTfR concentration in serum or plasma is directly proportional to erythropoietic activity and inversely proportional to tissue iron stores. Notably, sTfR is not significantly affected by the acute phase response, making it a more sensitive biomarker of iron deficiency than ferritin or transferrin saturation. On the other hand, since sTfR is sensitive to erythropoiesis, it is not an early indicator of iron deficiency.

Given that low ferritin is only an indicator of iron depletion and sTfR is an indicator of prior iron stores depletion, the use of these two biomarkers concurrently provides a fuller picture of iron status [108]. Therefore, several indicators that combine sTfR and ferritin have been proposed, such as the total body iron index (TBII), the sTfR/SF ratio, and the sTfR index [108,109]. While cutoffs have been suggested for these indexes, there is currently no standard methodology for the measurement of sTfR, and specific cutoffs are laboratory and assay-specific.

### 5.4 Erythrocyte protoporphyrin (EP)

Protoporphyrin (a precursor to hemoglobin) also binds with trace amounts of zinc forming zinc protoporphyrin (ZPP) in normal conditions. In the absence of sufficient iron, however, levels of ZPP rise in proportion to the depletion of iron stores. ZPP may be measured as free erythrocyte protoporphyrin (FEP) after a chemical extraction process that removes the zinc moiety. ZPP and FEP are interchangeable and collectively known as EP. Elevated EP is characteristic of iron-deficient erythropoiesis where EP > 30 μg/dL of whole blood or >70 μg/dL of red blood cells suggests a risk of iron deficiency in adults, according to CDC guidelines [99]. On the other hand, EP is also elevated in other processes of marked erythropoietic activity, such as in anemia of chronic disease, and is not strictly indicative of iron deficiency [109]. The concentration of EP is usually expressed as the amount relative to whole blood (μg/dL of whole blood) or red blood cells (μg/dL of packed red blood cells).

## 6 Dietary iron

Considering the intricate relationship between physiological iron imbalance and age-related pathologies, and since major regulation of iron levels occurs at the intestinal absorption level, it is evident that dietary iron intake must be finely coordinated. Dietary iron exists in two forms that can significantly differ in bioavailability [2]. Here we discuss these forms, and we provide suggestions on intake forms and levels of iron.

### 6.1 Nutritional forms of iron

#### 6.1.1 Dietary forms

**Fig. 3:**
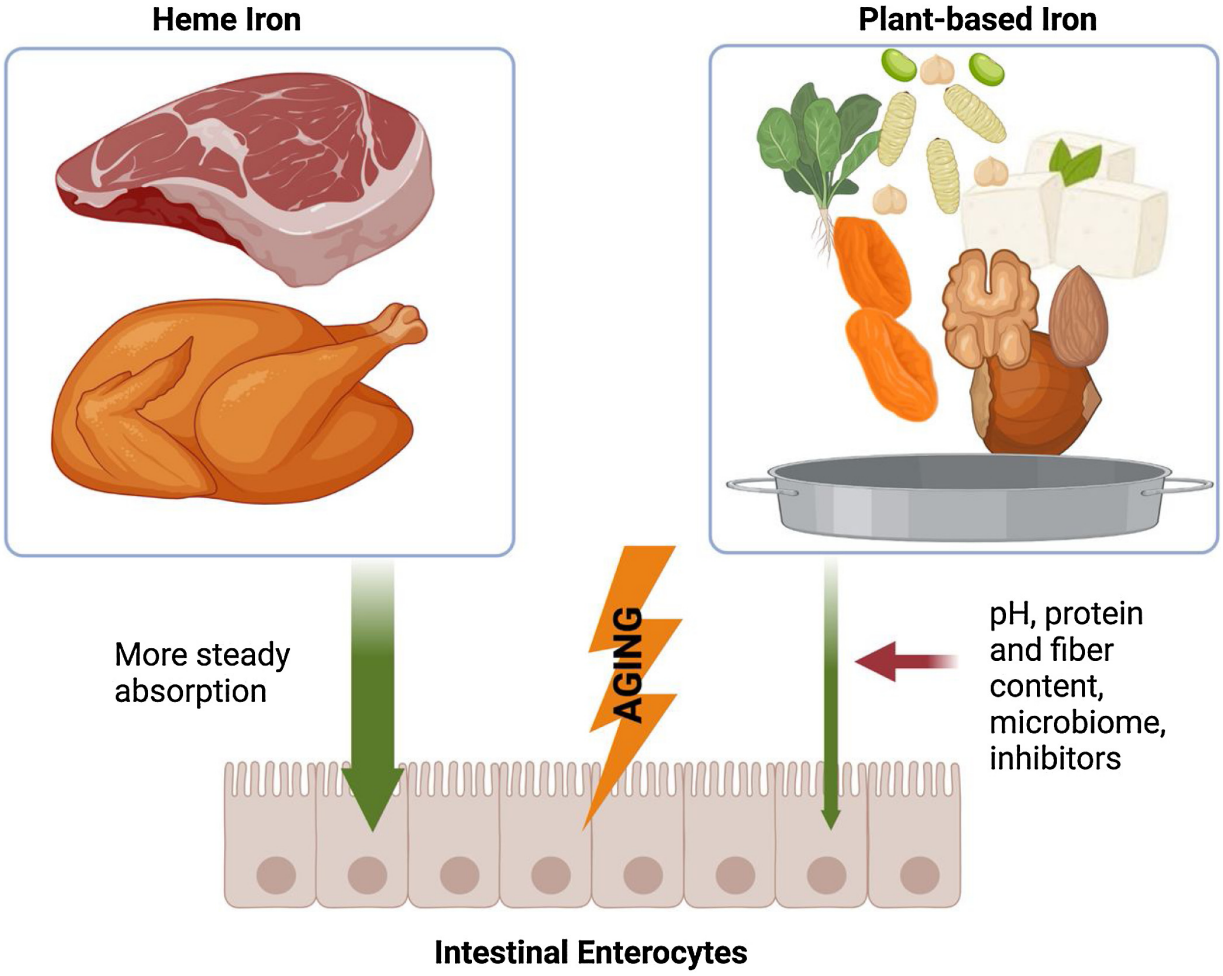
There are two forms of nutritional iron, animal based (heme iron) and plant-based iron. While heme iron is more stably and efficiently absorbed, plant based iron or non-heme iron absorption is less efficient and can be influenced by multiple factors including gut pH and microbiome. Age can have a profound effect on the efficiency of intestinal iron absorption of both forms.

Dietary iron exists in two forms, heme iron and non-heme iron (Fig. 3) [110]. Heme iron is found in animal products, where sources include red meat, poultry, fish, and shellfish [2], and accounts for more than 95% of functional iron in our body [111]. Heme iron from animal hemoglobin and myoglobin is easily absorbed by the body and is the most bioavailable form of iron [110]. This is significantly important in older adults where intestinal nutrient absorption can be jeopardized with age [112]. Non-heme iron (mostly plant-based) is present in various chemical forms, including ferritin and iron-sulfur clusters [1,113]. Non-heme iron, also called iron from vegetarian sources, is found in plant-based foods such as legumes, tofu, spinach, dried fruits, and fortified cereals (Table 3) [2,[114], [115], [116]]. and can also be found in eggs and dairy products [2,117].

**Table 3:** We have used the USDA website and the University of Rochester Medical Center Encyclopedia Nutrition Facts [151] to find the iron content in some of the plant-based foods.

Food	Serving size	Iron content	Equivalent iron RDI
Cooked Lentils	1 cup	6.6 mg	37%
Leafy greens (Ex: spinach, kale, collard greens)	1 cup	6.4 mg	36%
Nuts and seeds (Ex: pumpkin seeds, cashews, and almonds)	1 oz (28 g) – pumpkin seeds	4.2 mg	23%
Firm Tofu	Half a cup	3.4 g	>100%

#### 6.1.2 Dietary iron bioavailability

Previous studies have estimated iron bioavailability to be 14–18% for individuals having a mixed diet and 5–12% for vegetarians [118], thus, vegetarians and vegans have lower iron levels as compared to non-vegetarians [115,116]. Even though there are multiple plant-based sources of iron, the absorption of heme iron (animal-based) is generally more efficient and less affected by dietary factors [3]. Heme iron absorption is approximately 30%, while plant-based iron absorption is significantly less [3,118]. Iron absorption from plant-based iron sources should be in the ferrous state (Fe^2+^) to be absorbed and may be affected by multiple factors [110]. These include the duodenal pH and absorption inhibitors found in plants such as phytate and polyphenols, the presence of other nutrients such as ascorbic acid or vitamin C, body iron stores, and hepcidin levels [1,3,44]. Notably, the efficiency of heme iron absorption contributes significantly to maintaining physiological iron levels in individuals who consume a non-vegetarian diet [3]. While heme iron constitutes a smaller portion of dietary iron compared to non-heme iron, its higher bioavailability makes it an important contributor to overall iron balance in the body, yet it is less regulated by the body's iron stores compared to non-heme iron. [3]. Nevertheless, non-heme iron can also significantly contribute to overall iron intake [3]. Absorption of non-heme iron can be enhanced by consuming it with foods that are high in ascorbic acid or vitamin C (such as citrus fruits or bell peppers), which is an antioxidant that enhances iron absorption by reducing iron to its divalent state (Fe^2+^), facilitating its intestinal absorption via DMT1 [2,119,120].

#### 6.1.3 Other factors affecting dietary iron bioavailability

Additionally, calcium and dairy products can have an inhibitory effect on intestinal iron absorption [3]. The effect of protein on iron absorption, however, depends on the type of protein, such that pork protein can enhance iron absorption while soy protein isolate components can decrease absorption [3]. Dietary fibers are also known to reduce iron bioavailability [3,121,122]. Prebiotics, however, can improve heme iron absorption but not non-heme iron [123]. Short-chain fatty acids can enhance iron absorption [3,121,122]. Furthermore, certain types of cookware can contribute to the iron content of food. Cast iron cookware, for example, can leach small amounts of iron into food during cooking [124] potentially increasing food iron content by up to 20 times compared to cooking in stainless steel pots [125]. This can be beneficial for individuals who are at risk for iron deficiency or anemia.

### 6.2 Nutritional recommendations

#### 6.2.1 Recommended Dietary Allowance (RDA)

Adequate iron intake plays a pivotal role in healthy aging and longevity [2]. Currently, there are no guidelines for iron intake specific to older adults (>65 years old). The current RDA for iron intake in adults over 50 years, both male and female, is 8 mg per day [11]. The RDA is designed to meet the needs of 97% of the population [126]. Notably, an individual’s iron needs may vary depending on factors such as dietary habits, health status, and exercise levels [11]. Since age can affect the efficiency of intestinal iron absorption, and since older adults can have impaired iron homeostasis that can exacerbate chronic illnesses, it is particularly critical to maintain a balanced iron diet in older adults. This includes adequate amounts of both heme and non-heme iron sources without exceeding the Upper Tolerable Intake Level (UL) [127]. Consequently, it is recommended that older adults obtain dietary iron from a variety of foods including lean meats, lentils, beans, and dark green vegetables [128].

#### 6.2.2 Intervention strategies to reduce iron overload

Although iron chelators and phlebotomy are considered the first-line treatments for pathological iron overload, a healthy diet can be helpful for preventing and mitigating the condition [45,129]. Accumulating evidence supports the association between alcohol consumption (even at a moderate level) and the risk of iron overload, resulting in brain aging [130,131]. Notably, excessive iron particularly in older adults leads to multiple pathologies including insulin resistance [132]. Therefore, it is recommended that older adults with iron overload, regularly monitor their iron status while adhering to limited alcohol and limited iron absorption enhancer intake, as well as increased intake of iron inhibitors (tea, coffee, red wine) [133]. Importantly, vitamin C-rich foods should not be limited even in people with iron overload, since vitamin C deficiency is prevalent in older populations and patients with iron overload [134,135]. Moreover, since iron sequestration in the brain has been linked to neurodegeneration due to redox stress, an anti-inflammatory diet that is rich in vitamin E, and omega-3, and omega-6 polyunsaturated fatty acids could potentially alleviate the risk of neurodegeneration in older adults since it can slow the rate of brain iron accumulation [136].

#### 6.2.3 Intervention strategies for iron deficiency

Iron deficiency is another condition that is prevalent among older individuals. Iron supplementation is used to treat or prevent iron-deficiency anemia, which is usually characterized by low levels of hemoglobin. It is recommended that adults with anemia take between 60 and 200 mg of elemental iron (mainly ferrous sulfate) daily (with or without vitamin C) for the treatment of iron deficiency [105]. The absorption of oral iron from supplements can be limited by several factors, including the formulation (tablet type or liquid), the type of iron salt in traditional iron supplements, the presence of other dietary components including enhancers that can inhibit iron absorption (such as polyphenols, calcium, and phytates), and individual differences in gut health and microbiome composition [3,137,138]. As such, pairing iron supplements with vitamin C-rich foods, such as citrus fruits and cruciferous vegetables, could enhance the absorption of iron. However, the efficacy of using vitamin C supplements remains debatable as a randomized controlled trial noted similar effectiveness of iron supplementation (100 mg ferrous succinate) with and without vitamin C (200 mg) in patients with iron-deficiency anemia [139]. Furthermore, vitamin A and zinc might negatively impact iron absorption [140], which may limit the effectiveness of multivitamins/minerals for iron supplementation. The efficacy of iron supplementation can also be limited by the dosage used. High doses of iron can cause gastrointestinal side effects such as nausea, constipation, and diarrhea, which can reduce compliance with the supplement regimen [141]. Importantly, continuous iron supplementation has shown to be less effective since it can prompt upregulation of hepcidin and downregulation of intestinal iron transporters (i.e, DMT1 and FPN), and thus, alternate-day iron supplementation is proving to be more effective [142]. Furthermore, the duration of iron supplementation can also affect outcomes. Iron supplementation needs to be continued for a sufficient duration to achieve the desired effect, yet, compliance with long-term supplementation can be challenging.

It is crucial to mention that iron supplementation is not equally effective in all older adults. In addition to the interpersonal gastrointestinal physiological differences, genetic factors can also play a role in the effectiveness of iron supplementation, since individual genetic variations can affect iron metabolism, absorption, and utilization [143]. Additionally, some underlying conditions, such as inflammatory bowel disease, can interfere with iron absorption and utilization, limiting the effectiveness of iron supplementation [141,144]. Importantly, when taking iron supplements, it is critical to remember that the Tolerable Upper Intake Level (UL) for adults is 45 mg/day of iron, a level based on gastrointestinal distress as an adverse effect [2].

Anemia due to deficiencies in other vitamins including B12 and folic acid is also prevalent in older adults [36]. Therefore, supplementation with the lacking nutrients is an effective treatment in such cases. Furthermore, a wealth of evidence links obesity with iron deficiency [64,145,146]. Serum hepcidin and inflammatory cytokines (e.g., interleukin-6) are elevated in people with obesity as compared to those of normal weight, which can limit iron absorption, underscoring the importance of maintaining a healthy weight in older adults [147].

#### 6.2.4 Synopsis

Taken together, iron imbalance is common in older adults such that lower levels can lead to anemia and higher levels can lead to iron overload, both of which can exacerbate age-related pathological conditions. The regulation of iron homeostasis is a complex physiological process and can be impacted by both dietary intake and cellular removal processes. With age, these processes often become dysregulated, and thus older adults should monitor their iron intake and biological iron levels, using biomarkers of iron status on a regular basis. However, clear guidelines regarding dietary iron intake for older adults are currently lacking. More research is needed to better understand how interpersonal differences, including sex, underlying health conditions, genetic variabilities, and the gut microbiome can affect iron bioavailabity.

## 7 Conclusion

In conclusion, the impact of aging on iron homeostasis in older adults is multifaceted and extends to various aspects of cellular and systemic function. The downregulation of iron transporters, coupled with the influence of inflammaging on hepcidin levels, underscores the complexity of iron regulation in older adults. Other chronic diseases such as chronic kidney disease and heart failure can also affect iron metabolism and jeopardize iron homeostasis including absorption, utilization, and storage by a less well understood etiology [104,127,148]. Also, certain medications commonly prescribed to older adults, such as proton pump inhibitors and antacids, can interfere with iron absorption [149]. The impact of these medications on iron requirements and absorption is not well studied and therefore, customized guidelines for people taking these medications are still lacking. Additionally, interpersonal dissimilarities including genetic makeup, sex, lifestyle, and diet differences, are rarely considered, resulting in inaccurate guidelines for many people. Essentially, menopausal women are given the same iron RDA as men disregarding several factors such as hormonal changes. This intricate interplay between all these factors contributes to cellular iron sequestration, potentially giving rise to various pathologies.

Furthermore, compromised intestinal iron absorption in older adults poses a challenge to maintaining adequate dietary iron levels and limits the efficacy of traditional iron supplementation. Recognizing the potential risks associated with continuous iron supplementation, and to improve adherence and compliance, researchers advocate for alternative approaches, such as alternate-day dosing.

A strategic focus on mobilizing cellular iron emerges as a promising avenue for promoting efficient iron utilization in older adults. Physical exercise, especially activities that increase muscle mass, provides a beneficial intervention by enhancing the utilization of iron for myoglobin production and electron transport chain protein synthesis (enhanced oxidative phosphorylation for muscle respiration), thereby reducing cellular iron load. Moreover, lifestyle modifications, such as intermittent fasting and caloric restriction, offer additional strategies for mobilizing cellular iron. By reducing age-associated cellular iron load, these interventions have the potential to alleviate redox stress and mitigate cellular damage, thereby contributing to the prevention or amelioration of various age-related pathologies.

In essence, adopting a holistic approach that considers individual variations to customize specific interventions holds the potential for optimizing iron homeostasis among older adults. This approach not only tackles the difficulties arising from age-related alterations in iron regulation but also emphasizes the possibility of alleviating a wider range of age-associated pathologies by managing cellular iron dynamics. Achieving this goal necessitates the optimization of more precise and reliable methods for assessing iron status in older adults. Further research to address existing gaps in knowledge is imperative to provide updated and more accurate iron intake guidelines for adults in general and older adults in particular.

## 8 Authors’ contributions

Outline was set by RSZ and approved by CL, RTM and SA. Sections were assigned based on authors’ expertise and research interest. Sections 1, 2, and 7 (as well as the abstract) were assigned to RSZ, section 3 was assigned to MM and YL, sections 4.1 and 4.2 were assigned to CM and RSZ, section 4.3 to JJY, HSY and RSZ, section 5 to JT, and section 6 to AE and RSZ. RSZ revised and integrated all sections into a cohesive format, designed and crafted the figures, and finalized all other sections of the manuscript. Tables were prepared by RSZ, JT, and CM. TM revised and edited the paper, and finalized the formatting and referencing. JFC provided his professional feedback and comments on the content and revised the manuscript. CL, RTM, and SA provided feedback on the content, revised and edited the manuscript.

## Funding

This work was supported by the National Institute on Aging Training grantsT32AG062728for the work performed by Dr. Rola S. Zeidan and Dr. Taylor McElroy, T32AG049673for the work performed by Dr. Javier Tamargo, and T32AG020499for the work performed by Christian McLaren. This work has also been supported by the following grants: P30CA247796for Drs. Hyung-Suk Yoon and Jae Jeong Yang, and R01AG075136for Drs. Anton and Leeuwenburgh.

## Conflicts of interest

The authors declare no conflicts of interest.
